# Efficacy of phosphodiesterase-3 (PDE-3) inhibitors for the treatment of cerebral vasospasm in patients with aneurysmal subarachnoid haemorrhage (aSAH) admitted to the intensive care unit (ICU): a systematic review and meta-analysis protocol

**DOI:** 10.1136/bmjopen-2026-121153

**Published:** 2026-11-05

**Authors:** Mohammadi Maahtaab, Abubakr El Sheikh Idris, Breffni Smith, Pierce Geoghegan, Gerard F Curley, Natalie McEvoy

**Affiliations:** 1 School of Medicine, Royal College of Surgeons in Ireland (RCSI), Dublin, Ireland; 2 Beaumont Hospital, Dublin, Ireland; 3 Department of Library, Royal College of Surgeons in Ireland (RCSI), Dublin, Dublin, Ireland; 4 Department of Anaesthesia and Critical Care, Beaumont Hospital, Dublin, Ireland; 5 Department of Anaesthesia and Critical Care, RCSI, Dublin, Ireland; 6 Department of Anaesthesia and Critical Care, Royal College of Surgeons in Ireland, Dublin, Ireland

**Keywords:** Systematic Review, Meta-Analysis, NEUROSURGERY, INTENSIVE & CRITICAL CARE, Cerebral Hemorrhage

## Abstract

**Abstract:**

**Introduction:**

Aneurysmal subarachnoid haemorrhage (aSAH) complicated by cerebral vasospasm is a major contributor to delayed cerebral ischaemia (DCI), long-term neurological disability and mortality. Phosphodiesterase-3 (PDE3) inhibitors, such as milrinone and cilostazol, have shown potential to improve cerebral perfusion by reducing cerebral arterial vasoconstriction. However, there has been no systematic comparison of their effects in patients with aSAH. This review aims to assess the efficacy of PDE3 inhibitors, milrinone and cilostazol, in preventing and treating cerebral vasospasm in adult patients with aSAH.

**Methods and analysis:**

A systematic review and meta-analysis will be conducted in accordance with the Preferred Reporting Items for Systematic Reviews and Meta-Analyses (PRISMA) guidelines and the Cochrane Handbook. An initial search was conducted in July 2023 across MEDLINE, Embase, Web of Science and Scopus, with an updated search in August 2025. Eligible studies will include randomised controlled trials and observational studies with >15 patients evaluating the utility of milrinone or cilostazol in aSAH patients. Primary outcomes will be the incidence of and/or effect on cerebral vasospasm and DCI. Secondary outcomes will include mortality, neurological outcomes, intensive care unit and hospital length of stay, ventilator-free days, duration of vasopressor therapy and adverse events. Risk of bias will be assessed using the Cochrane RoB 2.0 tool, Risk Of Bias In Non-randomised Studies of Interventions tool and the Joanna Briggs Institute (JBI) checklist. Data will be synthesised using RevMan Web. The overall quality of evidence will be evaluated using the Grading of Recommendations Assessment, Development, and Evaluation approach.

**Ethics and dissemination:**

This systematic review does not require ethical approval as it synthesises previously published data. Findings will be submitted for publication in a peer-reviewed journal and disseminated at relevant conferences.

**Systematic review registration:**

The protocol is registered with the International Prospective Register of Systematic Reviews (PROSPERO): CRD42023444369.

**STRENGTHS AND LIMITATIONS OF THIS STUDY**

- A comprehensive search strategy developed with a clinical librarian ensures comprehensive evidence capture.
- Dual independent screening, risk of bias assessment using validated tools (RoB 2.0, Risk Of Bias In Non-randomised Studies of Interventions, JBI), and Grading of Recommendations Assessment, Development, and Evaluation evidence grading will strengthen the reliability of findings.
- The inclusion of both randomised controlled trials and observational studies may introduce clinical and methodological heterogeneity; narrative synthesis will be employed where meta-analysis is not appropriate.
- Restriction to English-language publications may limit the comprehensiveness of the evidence base.

## Introduction

 Cerebral aneurysms are characterised by abnormal dilation of cerebral arteries, primarily due to weakness in the vessel walls, with an estimated prevalence of around 3% in the adult population.^1^ Aneurysmal subarachnoid haemorrhage (aSAH), typically resulting from rupture of these aneurysms, presents as a medical emergency and remains a major contributor to mortality and long-term neurological disability.

One of the most significant complications of aSAH is cerebral vasospasm, which involves a pathological narrowing of the cerebral arteries, leading to compromised cerebral perfusion. This vasoconstriction is strongly implicated in the pathogenesis of delayed cerebral ischaemia (DCI), a condition defined by new-onset focal neurological deficits or a sustained drop in the Glasgow Coma Scale score of at least two points, lasting for at least 1 hour. Although not every case of cerebral vasospasm results in DCI, it remains the most significant predictor of its development.^2 3^

While the pathophysiology of cerebral vasospasm remains incompletely understood, several management strategies are used in isolation or combination for both treatment and prevention. These include induced hypertension for mean arterial pressure augmentation, prophylactic calcium channel blockers such as nimodipine and endovascular approaches including balloon angioplasty.^4–6^ Despite these advances, treatment strategies for vasospasm and DCI are heterogeneous and often centre-dependent, with variability in efficacy and safety. Pharmacological strategies targeting the underlying molecular mechanisms remain a promising adjunct.^7^

Phosphodiesterase (PDE) inhibitors represent one such class of agents. In the context of cerebral vasospasm, PDE3 inhibitors like milrinone and cilostazol prolong nitric oxide-mediated vasodilation and improve cerebral perfusion, thereby counteracting vasoconstriction and ultimately mitigating the occurrence of DCI.^8^

Although several individual studies have investigated the use of milrinone and cilostazol in aSAH, there has been no comprehensive synthesis of their relative efficacy in the management of cerebral vasospasm.^9–12^ To date, no systematic review has evaluated milrinone and cilostazol as PDE3 inhibitors under the same analytical framework, making it difficult to guide evidence-based clinical decisions regarding PDE3 inhibitor selection in aSAH.

This systematic review aims to address this gap by evaluating the efficacy of PDE3 inhibitors, milrinone and cilostazol, in both the treatment and prevention of cerebral vasospasm in aSAH, to help inform clinical decision-making and best practice guidelines on the use of PDE3 inhibitors in aSAH.

### Objectives

To assess the efficacy of PDE3 inhibitors (milrinone and cilostazol) in management of cerebral vasospasm and its effect on associated clinical outcomes in patients with aSAH admitted to the intensive care unit (ICU). The review will address three main comparisons:

- Milrinone versus placebo, standard care or an alternative agent.
- Cilostazol versus placebo, standard care or an alternative agent.
- Milrinone versus cilostazol, addressed by narrative comparison where evidence is available.

## Methods and analysis

This study is a protocol of the systematic review and meta-analysis of published articles. The protocol has been developed in accordance with the Preferred Reporting Items for Systematic Reviews and Meta-Analyses Protocols (PRISMA-P) statement (see online supplemental file 1).^13^ This systematic review was submitted for registration with International Prospective Register of Systematic Reviews (PROSPERO) in July 2023 (CRD42023444369).

Initially, the review aimed to include all classes of PDE inhibitors. However, to improve clarity and comparability across interventions, the scope was narrowed to focus exclusively on PDE3 inhibitors, milrinone and cilostazol. An initial database search was conducted in July 2023, followed by an updated search in August 2025, which yielded three additional eligible studies.

### Eligibility criteria

#### Study design

This systematic review will include all randomised controlled trials (RCTs), and prospective and retrospective observational studies, case series and pilot studies with more than 15 patients. Safety and feasibility studies with inadequate data, technical reports and study protocols will be excluded.

#### Participants

Adults (≥18 years) diagnosed with aneurysmal or non-traumatic subarachnoid haemorrhage (aSAH).

#### Intervention

Studies will be classified by treatment intent as follows:

- Prophylactic, defined as PDE3 inhibitor initiated before any clinical or radiological evidence of vasospasm or DCI.
- Therapeutic, defined as PDE3 inhibitor initiated after confirmation of vasospasm to improve further outcomes.

#### Comparator

Where available, comparators will be classified into two categories: placebo or standard care, and alternative vasodilator or comparison treatment agent such as magnesium sulphate. Coadministration of agents as part of standard care in both arms will be recorded as a cointervention rather than as a comparator.

### Outcomes

The following primary outcomes will be recorded:

- Angiographic (radiological) vasospasm: arterial narrowing on imaging irrespective of symptoms, classified by diagnostic modality (Digital Subtraction Angiography, CT angiography, transcranial Doppler) with the threshold for narrowing recorded. Incidence of angiographic vasospasm will be recorded for prophylactic category, and resolution/improvement of angiographic vasospasm will be recorded for therapeutic category.
- Symptomatic (clinical) vasospasm: neurological deterioration attributed to vasospasm and not defined by infarction, including outcomes reported as delayed ischaemic neurological deficit. Incidence of symptomatic vasospasm will be recorded for prophylactic category, and resolution of vasospasm will be recorded for therapeutic category.
- Cerebral infarction attributable to vasospasm or DCI: new infarction on CT or MRI within 6 weeks of ictus, not present on imaging performed 24–48 hours after aneurysm securing.

Secondary outcomes such as need for rescue endovascular therapy, neurological disability (measured by Modified Rankin Scale, Glasgow Outcome Scale, Glasgow Outcome Scale-Extended), mortality, length of ICU and hospital stay, number of ventilator-free days, duration of vasopressor therapy and adverse events will be recorded.

### Search strategy

A systematic literature search was conducted using MEDLINE, Embase, Web of Science and Scopus from inception to July 2023, and re-run in August 2025. Grey literature was identified using ClinicalTrials.gov and Google Scholar. Search terms included combinations of MeSH and free-text terms for ‘subarachnoid haemorrhage’, ‘cerebral vasospasm’, ‘phosphodiesterase inhibitors’, ‘milrinone’, and ‘cilostazol’. The search strategy was developed in consultation with a clinical librarian and finalised prior to the database search. Rayyan AI and EndNote were used for duplicate deletion and management of retrieved studies. A detailed example of the search strategy carried out on MEDLINE is provided in online supplemental file 2).

### Study selection

Title and abstract screening will be performed by two independent reviewers based on the eligibility criteria. Full-text articles of selected studies will be assessed for inclusion. Disagreements will be resolved by discussion or third-party adjudication. A PRISMA flow diagram will detail the screening process. Repeated reports from the same institution or registry are common in this literature, and a de-duplication step will therefore be performed during data extraction.

Inclusion criteria

- Peer-reviewed articles published in English language.
- RCTs, prospective and retrospective observational studies, case series with a minimum of 15 patients.
- Adults ≥18 years with aSAH.
- Studies evaluating PDE3 inhibitors (milrinone or cilostazol) in isolation or in conjunction with other treatments, by any route of administration.
- Administration of milrinone or cilostazol for cerebral vasospasm.

Exclusion criteria

- Case reports, expert opinions, reviews and meta-analyses.
- Animal or in vitro studies.
- Non-English language studies.
- Studies with patients <18 years.
- Studies that include traumatic SAH, ruptured arteriovenous malformations, dural venous fistulas or mycotic aneurysms.
- Studies without extractable or comparable outcome data.

### Study records and data management

#### Data extraction

A data extraction sheet was created and coded on MS Excel. Extracted variables include author name, year of publication, study design, sample size, baseline patient demographics, aSAH grading (Fisher, WFNS, Hunt and Hess), intervention details (drug, dose, route, duration, timing and intent of treatment), comparator, primary and secondary outcomes and conclusions. Additionally, definitions and thresholds of vasospasm and DCI will be recorded.

#### Risk of bias assessment

The risk of bias for RCTs will be assessed using the Cochrane Risk of Bias 2.0 tool.^14^ Non-randomised and observational studies will be appraised using the Risk Of Bias In Non-randomised Studies of Interventions (ROBINS-I) tool or the Joanna Briggs Institute (JBI) critical appraisal checklist as appropriate.^15 16^ Where possible, risk of bias assessments will be represented using a traffic light system, summary bar graphs, and summary tables separately for RCTs and observational studies.

#### Data synthesis

A summary of findings table will be created to summarise and compare the main findings of each study. Meta-analysis will be performed using RevMan Web where sufficient homogeneous data are available. Where homogeneous data are unavailable, findings will be reported narratively. Pooled estimates with 95% CIs will be calculated. Statistical heterogeneity will be evaluated using the I² statistic. I² >50% will be considered substantial heterogeneity. A random-effects model will be used for all meta-analyses, as substantial clinical and methodological diversity is anticipated across dose, route, timing of initiation, duration, comparator, case mix and severity, era and outcome definitions.

Subgroup and sensitivity analyses will be performed based on route of administration, combination therapy and treatment duration. Analyses will be stratified by treatment intent (prophylactic vs therapeutic), comparator category, study design and for vasospasm, type of vasospasm, diagnostic modality and threshold. Studies will not be pooled across these strata, and findings for milrinone and cilostazol will be synthesised and reported separately rather than combined. Where possible, an indirect narrative comparison between milrinone and cilostazol will be undertaken if a shared comparator is identified and the study populations are deemed to be sufficiently similar.

### Meta-bias(es)

Several potential meta-biases will be assessed in this review. Publication bias across studies will be evaluated using funnel plots where a sufficient number of studies are available (≥10 studies per meta-analysis), with formal statistical testing for asymmetry performed using Egger’s test. Where funnel plot asymmetry is detected, potential causes including publication bias, small-study effects and heterogeneity will be explored.

### Confidence in cumulative evidence

The overall quality of evidence for each outcome will be evaluated using the Grading of Recommendations Assessment, Development, and Evaluation framework. Ratings will be classified as high, moderate, low or very low based on study limitations, consistency, directness, precision and publication bias.

## Discussion

PDE3 inhibitors, specifically milrinone and cilostazol, have emerged as promising pharmacological agents due to their ability to enhance cyclic Adenosine Monophosphate signalling and prolong nitric oxide-mediated vasodilation. This pharmacological effect may counteract cerebral vasoconstriction and improve cerebral perfusion, potentially reducing the incidence of DCI.^17 18^ While several studies have investigated the effect of milrinone or cilostazol in this context individually, there remains a lack of systematic synthesis directly comparing these PDE3 inhibitors. Additionally, there is significant variation in the clinical settings, administration routes, outcome measures, and study designs, making it difficult to draw clear conclusions from the existing literature.

This systematic review and meta-analysis aims to address this gap by evaluating and comparing the efficacy of PDE3 inhibitors, milrinone and cilostazol, in the prevention and treatment of cerebral vasospasm and associated clinical outcomes in patients with aSAH. By focusing on PDE3 inhibitors, we aim to reduce clinical heterogeneity and facilitate meaningful evaluation under the same analytical framework, between interventions that share a common pharmacological mechanism. The review will also inform future research and help guide the development of best-practice guidelines and protocols for the management of cerebral vasospasm in aSAH.

## Ethics and dissemination

As this systematic review will only include data from previously published studies, formal ethical approval is not required. No individual patient data will be collected, accessed or analysed. Results will be written up and submitted for publication in a peer-reviewed open-access journal, with preference given to journals indexed in MEDLINE and PubMed. Findings will also be presented at relevant national and international critical care and neurosurgery conferences. This protocol has been prospectively registered with the PROSPERO (registration number: CRD42023444369).

## Supplementary material

10.1136/bmjopen-2026-121153 online supplemental file 1

10.1136/bmjopen-2026-121153 online supplemental file 2
