# Supplementary material for: Efficacy of phosphodiesterase-3 (PDE-3) inhibitors for the treatment of cerebral vasospasm in patients with aneurysmal subarachnoid haemorrhage (aSAH) admitted to the intensive care unit (ICU): a systematic review and meta-analysis protocol
Source: BMJ Open. 2026 Nov 5;16(11):e121153. doi: 10.1136/bmjopen-2026-121153 (PMC13756478; doi:10.1136/bmjopen-2026-121153)
Supplement: Supplementary file 1 [file bmjopen-2026-121153.reviewer_comments.pdf]

PEER REVIEW HISTORY

BMJ Open publishes all reviews undertaken for accepted manuscripts. Reviewers are asked to complete a checklist review form and are provided with free text boxes to elaborate on their assessment. These free text comments are reproduced below.

ARTICLE DETAILS

Title (Provisional)

Efficacy of Phosphodiesterase-3 (PDE-3) Inhibitors for the Treatment of Cerebral Vasospasm in Patients with Aneurysmal Subarachnoid Haemorrhage (aSAH) Admitted to the Intensive Care Unit (ICU): A Systematic Review and Meta-Analysis Protocol

Authors

Maahtaab, Mohammadi; El Sheikh Idris, Abubakr; Smith[3], Breffni; Geoghegan[4], Pierce; F. Curley, Gerard; McEvoy, Natalie

---

VERSION 1 - REVIEW

**Reviewer**	**1**
**Name**	**de Bruin , Jorg**
**Affiliation**	**Erasmus MC University Medical Center Rotterdam, Department of Vascular Surgery**
**Date**	**24-Jun-2026**
**COI**	**None**

This well written and clinically relevant protocol in the management of aneurysmal subarachnoid hemorrhage. The rationale is well explained, and the protocol is prospectively registered. The authors highlight the growing interest in PDE-3 inhibitors as adjunctive therapy for cerebral vasospasm. The objective, however, appears not clear, throughout the manuscript the authors describe directly comparing milrinone with cilostazol. Given that most available studies compare either agent with standard care rather than with each other, it is unclear whether the review will assess treatment efficacy with standard of care and/or compares both medications with each other. Furthermore, substantial clinical heterogeneity is anticipated due to differences in treatment protocols, timing of treatment and study designs. The protocol currently provides insufficient detail how these issues will be addressed apart from statistics.

1. What is the primary hypothesis of the review? Is the objective to evaluate PDE3 inhibitors versus standard care, compare milrinone versus cilostazol and/or both? Please clarify.

2. Given that few studies directly compare milrinone and cilostazol, how do the authors intend to perform a valid comparative assessment? Please explain.

3. How will the authors address differences in timing and route of administration for both medications? Please answer.

4. Will the authors make a difference in treatment regarding prophylactic treatment and established vasospasm treatment? Please clarify.

5. Will the authors pool randomised and observational studies within same analysis? Can the authors account for study design within this analysis? Please explain.

6. Will a fixed effect or a random effect model be used and what is the rationale for this? Please answer.

7. How will overlapping patient cohorts from similar institutions and/or registries identified and managed within the analysis? Please clarify.

8. How will the different definitions of cerebral vasospasm amongst the studies (clinical vs different imaging modalities) be harmonized for the meta-analysis? Please explain.

VERSION 1 - AUTHOR RESPONSE

We would like to thank you and the reviewers for the feedback on our manuscript. We have taken all comments on board and we believe the paper has now substantially improved as a result. Please see our response to reviewer comments below:

**Reviewer comment**	**Author response**	**Where amended**
General<br>The objective appears not clear; throughout the manuscript the authors describe directly comparing milrinone with cilostazol. Given that most available studies compare either agent with standard care rather than with each other, it is unclear whether the review will assess treatment efficacy with standard of care and/or compares both medications with each other. Furthermore, substantial clinical heterogeneity is anticipated due to	We thank Dr de Bruin for a careful and constructive appraisal, and we accept both points.<br>On the objective, the description of a direct comparison was an overstatement and has been corrected throughout, including in the abstract, Strengths and Limitations box, Introduction, Objectives and Discussion. The agent-specific comparisons against placebo or standard care are now stated as primary, and the between-agent comparison as secondary if evidence permits.<br>On heterogeneity, we accept that the original protocol addressed it almost entirely through the statistical plan. The revised protocol now addresses it at three levels, of which the analytic level is the last rather than the first:<br>● Eligibility and classification: studies will be classified prospectively by treatment intent (prophylactic and therapeutic), and furthermore into agent, route and comparator category, and these categories are not pooled with one another (Comments 1, 3, 4).<br>● Data extraction and outcome harmonisation: regimen, timing, duration and co-interventions are extracted, outcome definitions are recorded verbatim, and a de-duplication step is added (Comments 7, 8).	Throughout<br>Methods and analysis

differences in treatment protocols, timing of treatment and study designs. The protocol currently provides insufficient detail how these issues will be addressed apart from statistics.	● Analysis: synthesis is stratified by design, with pre-specified rules for when pooling will not be attempted (Comments 5, 6).	
1.<br>What is the primary hypothesis of the review? Is the objective to evaluate PDE3 inhibitors versus standard care, compare milrinone versus cilostazol and/or both? Please clarify.	We accept that the Objectives section did not distinguish between these questions, and have amended it. The primary objective is to evaluate each agent against placebo or standard care; comparison between the two agents would be secondary where evidence permits, i.e., where a common comparator is identified and the study population and treatment intent is deemed to be sufficiently similar.<br>The revised Objectives are as follows: To assess the efficacy of PDE3 inhibitors (milrinone and cilostazol) in management of cerebral vasospasm and its effect on associated clinical outcomes in patients with aSAH admitted to the ICU. This would be addressed across three comparisons: milrinone versus placebo, standard care or an alternative agent; cilostazol versus placebo standard care or alternative agent; and milrinone versus cilostazol, addressed narratively where evidence permits.<br>We have also confirmed in the eligibility criteria that the review is restricted to the selective PDE3 inhibitors milrinone and cilostazol, and does not extend to non-selective phosphodiesterase inhibitors or to inhibitors of other PDE isoforms.	Objectives, lines 116 - 122;<br>Data Synthesis: lines 251-253
2.<br>Given that few studies directly compare milrinone and cilostazol, how do the authors intend to perform a valid comparative assessment? Please explain.	We accept this criticism in full, and our scoping of the literature at the point of full text screening showed that, further to the absence of sufficient studies directly comparing milrinone and cilostazol, the two agents have largely been evaluated against different comparators. Cilostazol has mainly been assessed as oral prophylaxis compared with standard care or placebo, while milrinone has mainly been used as rescue therapy for established vasospasm, often in uncontrolled series or against an alternative agent or regimen<br>As a result, findings for the two agents will be synthesised and reported separately. Where both agents are reported for the same outcome, findings will be presented side by side with their populations, regimens, comparators and outcome definitions, and interpreted narratively.<br>An indirect narrative comparison will be undertaken if a shared comparator is identified and the study populations are judged sufficiently similar. The between-agent comparison will, thus, consist of a structured narrative synthesis alongside a side-by-side summary of populations, regimens, comparators, outcome definitions, effect estimates and GRADE certainty,<br>We have also restructured the Objectives section into three categories: milrinone versus standard care or placebo, cilostazol versus standard care or placebo, and milrinone versus cilostazol where evidence permits.	Objectives, lines 116 - 122;<br>Data Synthesis: lines 251-253

3<br>How will the authors address differences in timing and route of administration for both medications? Please answer.	Extraction fields have been expanded to capture, for each study arm, the agent, route, dose and dose range, timing of initiation relative to ictus and relative to the diagnosis of vasospasm or DCI, duration, criteria for escalation and cessation, and co-interventions including nimodipine, induced hypertension, magnesium, statins and endovascular rescue.<br>Route is a substantial source of diversity within the milrinone literature. Subgroup analyses will be pre-specified for route, timing of initiation and duration, reported with the number of contributing studies, and interpreted as exploratory using the ICEMAN criteria.<br>We also wish to acknowledge one constraint the question: cilostazol is an oral agent and milrinone a parenteral or intrathecal one, so route is confounded with choice of agent and route-specific effects cannot be separated from agent-specific effects. Any apparent difference between agents may reflect route, timing or population rather than pharmacology, and we will acknowledge this in our final systematic review manuscript	Data extraction, lines 225-226;<br><br>Data Synthesis, lines 242-251.
4<br>Will the authors make a difference in treatment regarding prophylactic treatment and established vasospasm treatment? Please clarify.	Yes, treatment intent and timing will be a primary stratifying variable, and the categories are not pooled with one another.<br>Two categories are defined: prophylactic (initiated before any clinical or radiological evidence of vasospasm or DCI and therapeutic (initiated after confirmation of vasospasm).<br>Where a study includes more than one intent, strata will be extracted separately where the data permit. Studies in which timing cannot be determined will not contribute to pooled analyses.<br>Because the intents pose different clinical questions, outcomes relevant to therapeutic intent will be recorded; for instance, resolution or angiographic improvement of established vasospasm, incidence of DCI, and requirement for rescue endovascular therapy will be recorded for therapeutic management, and incidence of cerebral vasospasm and DCI will be recorded for prophylactic management.	Intervention, lines 165-169
5<br>Will the authors pool randomised and observational studies within same analysis? Can the authors account for study design within this analysis? Please explain.	No. Randomised and non-randomised studies will not be pooled within the same analysis, and synthesis will be stratified by design in accordance with Chapter 24 of the Cochrane Handbook<br><br>Where two or more randomised trials are available for a comparison and outcome, the randomised evidence constitutes the primary analysis. Non-randomised studies will be synthesised separately using adjusted effect estimates where reported, with adequacy of adjustment judged during ROBINS-I assessment. GRADE is applied separately to each body of evidence, with non-randomised evidence beginning at low certainty.<br>We understand that stratification may leave few studies per stratum, however where fewer than three studies of a given design contribute to a comparison and outcome, no pooled estimate will be produced for that stratum and the studies will be tabulated with individual effect estimates and synthesised narratively.<br><br>Uncontrolled single-arm series (which form a substantial part of the milrinone literature) will not contribute to comparative estimates under any circumstance, and will contribute to proportion meta-analysis only for adverse event outcomes with clearly defined outcomes.	Risk of Bias assessment lines 257-258

6<br>Will a fixed effect or a random effect model be used and what is the rationale for this? Please answer.	We anticipate a random effects analysis will be used as a random effects assumption is less restrictive that a fixed effects assumption and reflects the variation or heterogeneity in the true effects estimated by each trial. To allow for differences in study characteristics we anticipate such as dose, duration, timing etc, a random effects model is considered to be more appropriate.	Data synthesis, lines 266-268
7<br>How will overlapping patient cohorts from similar institutions and/or registries identified and managed within the analysis? Please clarify.	We agree this is a material risk in this literature, where several centres have published repeatedly on the same institutional protocol and where national registry data are also available.<br>Potential overlap will be flagged on the basis of institution and city, recruitment period, trial or registry identifier, research ethics approval number, author overlap, and correspondence between sample size and baseline characteristics. Where overlap is suspected but cannot be resolved from the published reports, corresponding authors will be contacted and the outcome recorded.<br>Reports judged to describe the same or overlapping patients will be grouped into a study cluster, and one report per cluster will contribute to any given analysis, selected based on factors such as largest number of non-overlapping participants; the most complete reporting of the outcome under analysis; the longest follow-up; the most recent publication date.	Study selection, lines 221-225
8<br>How will the different definitions of cerebral vasospasm amongst the studies (clinical vs different imaging modalities) be harmonized for the meta-analysis? Please explain.	Our revised manuscript now categorically separates and defines two different types of vasospasm: angiographic (radiological) vasospasm, defined as arterial narrowing on imaging irrespective of symptoms; symptomatic (clinical) vasospasm, defined as neurological deterioration attributed to vasospasm and not defined by infarction; and cerebral infarction attributable to vasospasm or DCI. These will be analysed separately and will not be combined into a composite.<br><br>Angiographic vasospasm will be stratified by diagnostic modality and pooled only within modality class, with the threshold for arterial narrowing entered as a subgroup, since thresholds in this literature range from greater than 25 per cent to greater than 50 per cent narrowing and do not identify the same patients.<br>Some studies report delayed ischaemic neurological deficit and delayed cerebral ischaemia as separate entities, the former denoting symptomatic vasospasm and the latter the resulting infarction, whereas the Vergouwen [1] consensus definition combines clinical deterioration and infarction under the single term DCI. Each reported outcome will therefore be mapped onto the construct it measures rather than onto the label it carries.<br>Studies may report angiographic outcomes per vasospastic arterial segment or per vascular territory rather than per patient; the denominator will be recorded and segment-level and patient-level data will not be pooled.<br>Harmonisation will also take account of treatment intent, as the same outcome maybe measured differently in the two settings. For prophylactic studies, the incidence of angiographic and symptomatic vasospasm will be recorded; for therapeutic studies, resolution or improvement of	Outcomes, lines 177-199;<br><br>Data extraction, line 251.

	established vasospasm. These will not be combined, as an incidence and a resolution rate are not the same quantity<br>Definitions and thresholds will be extracted studies reporting an outcome without a stated definition will not contribute to pooled analyses	

We hope that the revisions address the reviewer's comments satisfactorily. We are grateful for the constructive feedback, which we believe has strengthened the protocol, and we would be happy to make further amendments if required.

References

1. Vergouwen MDI, Vermeulen M, van Gijn J, et al. Definition of delayed cerebral ischemia after aneurysmal subarachnoid hemorrhage as an outcome event in clinical trials and observational studies. Stroke. 2010;41(10):2391-5.

VERSION 2 - REVIEW

Reviewer 1

Name de Bruin , Jorg

Affiliation Erasmus MC University Medical Center Rotterdam, Department of Vascular Surgery

Date 15-Sep-2026

COI

The authors have significantly improved the manuscript.
