# Supplementary material for: Efficacy of phosphodiesterase-3 (PDE-3) inhibitors for the treatment of cerebral vasospasm in patients with aneurysmal subarachnoid haemorrhage (aSAH) admitted to the intensive care unit (ICU): a systematic review and meta-analysis protocol
Source: BMJ Open. 2026 Nov 5;16(11):e121153. doi: 10.1136/bmjopen-2026-121153 (PMC13756478; doi:10.1136/bmjopen-2026-121153)
Supplement: Supplementary file 3 [file bmjopen-16-11-s002.docx]

## Search Keywords

| **Intervention** | **AND** | **Population** |
| --- | --- | --- |
| **OR** |  | **OR** |
| Phosphodiesterase inhibitor |  | Subarachnoid hemorrhage |
| PDE inhibitor |  | Subarachnoid haemorhage |
| Phosphodiesterase 3 inhibitor |  | Aneurysmal Subarachnoid hemorrhage |
| Phosphodiesterase 4 inhibitor |  | Aneurysmal Subarachnoid haemorrhage |
| Phosphodiesterase 5 inhibitor |  | Ruptured cerebral aneurysm |
| Cilostazaol |  | aSAH |
| Papaverine |  | SAH |
| Tadalafil |  |  |
| Sildenafil |  |  |
| Milrinone |  |  |
| PDE III inhibitor |  | Cerebral vasospasm |
| PDE IV inhibitor |  |  |
| PDE V inhibitor |  |  |

Database: MEDLINE via PubMed

Date last searched: August 2025

Date limits: None applied (searched from inception)

Language limits: English

.

# Boolean search carried out on PubMed:

# Block 1

(((((("Milrinone"[Mesh]) OR ("Cilostazol"[Mesh])) OR (((("Papaverine"[Mesh])) OR "Tadalafil"[Mesh]) OR "Sildenafil Citrate"[Mesh])) OR ("Dipyridamole"[Mesh])) OR ("Phosphodiesterase Inhibitors"[Mesh] OR "Phosphodiesterase 3 Inhibitors"[Mesh] OR "Phosphodiesterase Inhibitors" [Pharmacological Action] OR "Phosphodiesterase 4 Inhibitors"[Mesh] OR "Phosphodiesterase 5 Inhibitors"[Mesh])) OR ((((((((((((((((("milrinone"[All Fields]) OR ("cilostazol"[All Fields])) OR ("papaverine"[All Fields])) OR ("tadalafil"[All Fields])) OR ("sildenafil"[All Fields])) OR ("dipyridamole"[All Fields])) OR ("phosphodiesterase inhibitors"[All Fields])) OR ("phosphodiesterase 3 inhibitors"[All Fields])) OR (Phosphodiesterase 4 Inhibitors)) OR ("phosphodiesterase 5 inhibitors"[All Fields])) OR ("pde inhibitor"[All Fields])) OR ("pde iii inhibitor"[All Fields])) OR ("pde iv inhibitor"[All Fields])) OR ("pde v inhibitor"[All Fields])) OR ("pde 3 inhibitor"[All Fields])) OR ("pde 4 inhibitor"[All Fields])) OR ("pde 5 inhibitor"[All Fields])))

# Block 2

((((((((((("subarachnoid hemorrhage"[All Fields]) OR ("subarachnoid haemorrhage"[All Fields])) OR ("aneurysmal subarachnoid hemorrhage"[All Fields])) OR ("ruptured cerebral aneurysm"[All Fields])) OR ("sah"[All Fields])) OR ("asah"[All Fields])) OR ("aneurysmal subarachnoid haemorrhage"[All Fields])) OR ("Subarachnoid Hemorrhage"[Mesh]))) OR ("Vasospasm, Intracranial"[Mesh])) OR ("cerebral vasospasm"[All Fields]))

Combined Search

(1) AND (2)

(((((("Milrinone"[Mesh]) OR ("Cilostazol"[Mesh])) OR (((("Papaverine"[Mesh])) OR "Tadalafil"[Mesh]) OR "Sildenafil Citrate"[Mesh])) OR ("Dipyridamole"[Mesh])) OR ("Phosphodiesterase Inhibitors"[Mesh] OR "Phosphodiesterase 3 Inhibitors"[Mesh] OR "Phosphodiesterase Inhibitors" [Pharmacological Action] OR "Phosphodiesterase 4 Inhibitors"[Mesh] OR "Phosphodiesterase 5 Inhibitors"[Mesh])) OR ((((((((((((((((("milrinone"[All Fields]) OR ("cilostazol"[All Fields])) OR ("papaverine"[All Fields])) OR ("tadalafil"[All Fields])) OR ("sildenafil"[All Fields])) OR ("dipyridamole"[All Fields])) OR ("phosphodiesterase inhibitors"[All Fields])) OR ("phosphodiesterase 3 inhibitors"[All Fields])) OR (Phosphodiesterase 4 Inhibitors)) OR ("phosphodiesterase 5 inhibitors"[All Fields])) OR ("pde inhibitor"[All Fields])) OR ("pde iii inhibitor"[All Fields])) OR ("pde iv inhibitor"[All Fields])) OR ("pde v inhibitor"[All Fields])) OR ("pde 3 inhibitor"[All Fields])) OR ("pde 4 inhibitor"[All Fields])) OR ("pde 5 inhibitor"[All Fields])))

AND

((((((((((("subarachnoid hemorrhage"[All Fields]) OR ("subarachnoid haemorrhage"[All Fields])) OR ("aneurysmal subarachnoid hemorrhage"[All Fields])) OR ("ruptured cerebral aneurysm"[All Fields])) OR ("sah"[All Fields])) OR ("asah"[All Fields])) OR ("aneurysmal subarachnoid haemorrhage"[All Fields])) OR ("Subarachnoid Hemorrhage"[Mesh]))) OR ("Vasospasm, Intracranial"[Mesh])) OR ("cerebral vasospasm"[All Fields])
